# Visits to Private Asylums

**Published:** 1904-12-03

**Authors:** 


### VISITS TO PRIVATE ASYLUMS.

By Our Representative.

### ASHBROOK HALL, HOLLINGTON.

Hollington is a village of 800 inhabitants about two and a half miles north-west of Hastings, and a motor-omnibus leaves the Albert Memorial for the village every twenty minutes or so. Ashbrook Hall is only a few minutes' walk from the Hollington terminus. We pass the church of St. Leonard on the left and take a quiet country road on the right. The Hall may be known by one of those ugly fire-escape staircases with which so many of our private asylums have lately been disfigured; but excepting this feature it has neither externally nor internally anything suggestive of an institution. In fact it is not one, but is merely a private house with unlocked doors, cheerful rooms, and pleasant surroundings; a house where six ladies can be received and can find a comfortable home within its walls. There are drawing-rooms and private sitting-rooms, and each patient has a bedroom to herself and her own nurse. The bedrooms are unusually large, airy, bright, and comfortably furnished. The proprietress takes her meals with the patients, and so emphasises the home-like arrangements of the asylum. The grounds extend to several acres, and there are pleasant walks and large lawns, and when the wind is blowing from the sea, only two miles away, the invigorating breezes can be enjoyed in the garden. In summer tents are put up and the patients often have their meals out of doors. Mrs. Hitch, the present proprietress, has held the license for more than twenty years. Her late husband was at one time well known for the interest he took in all matters connected with the insane, and was for many years physician to the Asylum for the county of Gloucester. Miss Adams is now co-licensee with Mrs. Hitch. The house nearly always contains its full complement of patients, and as might be expected the fees are somewhat high, but not higher than the accommodation justifies.

